# Unity and disunity in evolutionary sciences: process-based analogies open common research avenues for biology and linguistics

**DOI:** 10.1186/s13062-016-0145-2

**Published:** 2016-08-20

**Authors:** Johann-Mattis List, Jananan Sylvestre Pathmanathan, Philippe Lopez, Eric Bapteste

**Affiliations:** 1 CRLAO/EHESS, 2 rue de Lille, Paris, 75007 France; 2 Equipe AIRE, UMR 7138, Laboratoire Evolution Paris-Seine, Université Pierre et Marie Curie, 7 quai St Bernard, Paris, 75005 France

**Keywords:** Process-based analogies, Language evolution, Protein assembly, Word formation, Lateral transfer, Constructive neutral evolution, Similarity networks, Incomplete lineage sorting

## Abstract

**Background:**

For a long time biologists and linguists have been noticing surprising similarities between the evolution of life forms and languages. Most of the proposed analogies have been rejected. Some, however, have persisted, and some even turned out to be fruitful, inspiring the transfer of methods and models between biology and linguistics up to today. Most proposed analogies were based on a comparison of the research *objects* rather than the *processes* that shaped their evolution. Focusing on *process-based analogies*, however, has the advantage of minimizing the risk of overstating similarities, while at the same time reflecting the common strategy to use processes to explain the evolution of complexity in both fields.

**Results:**

We compared important evolutionary processes in biology and linguistics and identified processes specific to only one of the two disciplines as well as processes which seem to be analogous, potentially reflecting core evolutionary processes. These new *process-based analogies* support novel methodological transfer, expanding the application range of biological methods to the field of historical linguistics. We illustrate this by showing (i) how methods dealing with incomplete lineage sorting offer an introgression-free framework to analyze highly mosaic word distributions across languages; (ii) how sequence similarity networks can be used to identify composite and borrowed words across different languages; (iii) how research on partial homology can inspire new methods and models in both fields; and (iv) how constructive neutral evolution provides an original framework for analyzing convergent evolution in languages resulting from common descent (*Sapir’s drift*).

**Conclusions:**

Apart from new analogies between evolutionary processes, we also identified processes which are specific to either biology or linguistics. This shows that general evolution cannot be studied from within one discipline alone. In order to get a full picture of evolution, biologists and linguists need to complement their studies, trying to identify cross-disciplinary and discipline-specific evolutionary processes. The fact that we found many process-based analogies favoring transfer from biology to linguistics further shows that certain biological methods and models have a broader scope than previously recognized. This opens fruitful paths for collaboration between the two disciplines.

**Reviewers:**

This article was reviewed by W. Ford Doolittle and Eugene V. Koonin.

**Electronic supplementary material:**

The online version of this article (doi:10.1186/s13062-016-0145-2) contains supplementary material, which is available to authorized users.

## Background

Biological objects on Earth have been evolving for billions of years. The origin of language evolution dates back to only about 200 000 years ago. The specific aspects of the evolution of life forms and the evolution of languages are traditionally investigated by the disciplines of evolutionary biology and historical linguistics. The research objects of the two disciplines differ greatly. Biology deals with substantial objects, that is, objects with a concrete physical manifestation. Languages, on the other hand, are ‘products of the human mind’ ([1], p. 144). They are intellectual objects ([2], p. 72), that is, objects whose manifestation is based on the interaction between humans. They are realized physically, be it when they are spoken or written down, but their realization is dependent on the existence of individuals who speak and understand them, and in this way, language systems are constantly being reconstructed by new speakers who learn them [3].

**Fig. 1:**
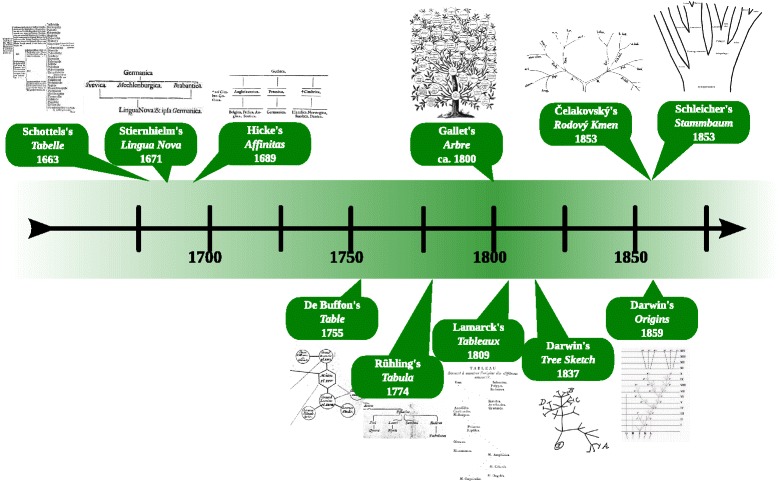
Timeline of early tree- and network diagrams in linguistics (*top*) and biology (*bottom*). Schottel’s branching table of Germanic languages from 1663 is the earliest we could identify. The three following early diagrams in linguistics by Stiernhielm (1671) [7], Hickes (1689), [9], and Gallet (1800) [8] all contain reticulation, real trees only start with Ćelakovský and Schleicher (1853) [4, 15]. The situation is similar in biology, where the two schemas by Leclerc De Buffon (1755) [12] and Rühling (1774) [13] allow for reticulation, in contrast to Lamarck (1809) [17] and Darwin (1837, 1859) [5, 16]

Similar models have been developed independently in the history of both disciplines. Both biologists and linguists have a long tradition of using trees to model diversification by a genealogy. Trees were independently popularized by August Schleicher (1821–1868) in 1853 [4] and Charles Darwin (1809–1882) in 1859 [5]. Both fields also share a more recent tradition of using networks to capture reticulation, although early network models of languages [6–9] (see [10, 11]) and life forms [12, 13] (see [14]) even predate the classical family trees [4, 5, 15–17] (see [10, 14, 18], and Fig. 1). Some processual similarities are also reflected in the methods independently developed and applied in both disciplines, such as, for example, cladistic approaches and alignment analyses. In linguistics, approaches for subgrouping based on shared innovations (or shared derived characters) date back to the end of the 19th century ([19], p. 24). In biology they were independently developed in the middle of the 20th century [20]. At about the same time, first approaches to numerical tree reconstruction based on distance data can be found in both disciplines [21, 22]. Although only sporadically applied and never fully automatized, early examples in which linguists aligned corresponding sounds in multiple homologous words can already be found in the early 20th century [23–25]. In biology, automatic methods for sequence alignment were developed from 1970 onwards soon after the rise of molecular biology [26–28]. Both biologists and linguists also struggle with common epistemological limitations, since the processes they investigate lie in the past, which is why uniformitarianism, the assumption that the processes observed today do not differ much from the processes which happened in the past ([29], p. 165), still plays an important role in biology and linguistics [30–32].

Apart from similar models and methods developed independently, there was and is also a considerable amount of explicit transfers between the two disciplines. An early example is the intimate intellectual exchange on Darwin’s evolutionary theory and its implications for the study of languages between the biologist Ernst Haeckel (1834–1919) and the linguist August Schleicher (1821–1861) [33]. According to this correspondence, it was Haeckel who brought Schleicher’s attention to the work of Darwin. Schleicher was deeply impressed by the similarities of the research objects in such different domains ([34], p. 6). He emphasized, however, also that these parallels would only hold for the essential features, not for the details ([33], p. 29). Haeckel, in turn, took inspiration from Schleicher’s language tree diagrams to promote evolutionary tree drawing in biology ([10], p. 300).

In the 20th century, especially the early work on genetics, not long after the correct modelling of the structure of DNA by Watson and Crick [35], was characterized by a strong linguistic influence. This is reflected in the multitude of linguistic terms, like ‘alphabet’ and ‘word’ [36] or ‘translation’ [37], which were used to describe biological phenomena in the biological domain [38]. While, as indicated by Eugene V. Koonin (one of the reviewers of this manuscript), the majority of these terms reflected mere metaphors of which only a minority became later integrated into the standard terminology of biology (see also [39]), we can also find examples for the explicit transfer of linguistic methods and theories to the biological domain. Thus, up to today, the theory of formal grammar [40] plays an important role in addressing certain problems in bioinformatics [41], like RNA folding and protein structure analysis, and it is not uncommon for biological textbooks on sequence comparison to also include a chapter on formal grammars ([42], pp. 233-259). This influence is not restricted to classical models of grammar [43]. Advanced models, like *tree adjoining grammar*, have likewise been used for RNA structure prediction [44], and inherently linguistics methods, like methods for document prediction, have been successfully applied for the task of protein classification [45]. During the last twenty years the direction of interdisciplinary transfer has turned, and many methods originally designed for applications in evolutionary biology have been applied to linguistic data. These include algorithms for phylogenetic reconstruction [46, 47], phylogenetic network approaches [48–52], multiple sequence alignment [53–55], and homolog identification [55, 56].

In the following, we will argue that these transfers can be further enhanced. By shifting from the comparison of research *objects* to the comparison of *processes* affecting the research objects in the disciplines, wrong analogies due to an exaggeration of similarities and a neglection of differences can be avoided. At the same time, the identification of important processes, common to language and biological evolution, can give rise to new, potentially fruitful analogies. For linguistics, these transfers offer new theoretical and practical ways to explain the mosaic distributions of words across related and unrelated languages, with and without invoking processes of lateral transfer. A new analogy between the process of word formation in linguistics and protein assembly in biology offers a fresh perspective on the idea of a *protein grammar* [57] and can inspire new methods and models in both fields. Invoking a system perspective can further help to demystify the phenomenon of convergent evolution in languages resulting from common descent.

## Process-based analogies

The striking similarities between biological and language evolution opt for a systematic investigation of analogies in the two disciplines. Such an investigation may cumulate in a program whose objectives would be (a) to investigate the isomorphy of processes, methods, and models in the two disciplines, (b) to foster the development of models lacking in either of the disciplines, and (c) to reduce the duplication of effort. Such a program, very close to the one proposed by the Society for General Systems Research in 1954 (as reported by ([58], p. 13)), would further ‘promote the unity of evolutionary science through improving communication among specialists’ (adapted from ([58], p. 13)). A multitude of analogies between biology and linguistics has been proposed in the past 200 years [59]. Languages have been compared with organisms ([60], p. 16f), species [61], microbes [49, 50], mutualist symbionts [62], and populations [63]. Words have been compared with cells ([33], p. 23f), amino-acids [64], codons [65, 66] and genes [61]. Sounds (phonemes) have been compared with nucleic bases [65, 67] and atoms [64]. Only a small amount of these analogies has received broader attention, many have been rejected quickly after they were first proposed, and only recently, an explicit transfer of methods and models has been initiated [68].

**Fig. 2:**
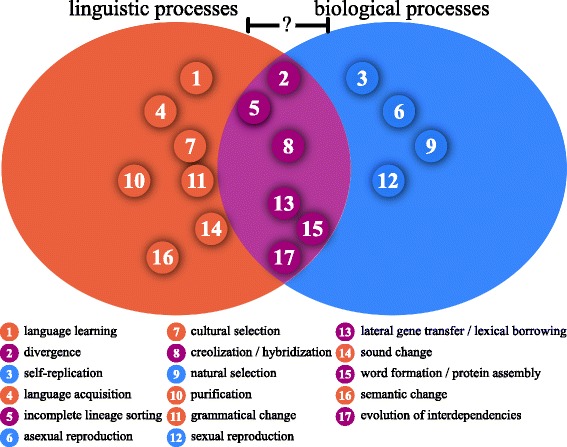
Contrasting purely linguistic, purely biological, and analogous processes in linguistics and biology. For Process-Based Analogies, we contrast the biological term with the linguistic term, if both disciplines address the processes in their terminology. See the text for further clarification

We find two main reasons why the majority of analogies that have been proposed between biology and linguistics have not turned out to be fruitful on the long run. First, most of the proposed analogies are object-based, taking the research objects as their main comparandum. Second, given the different media in which the research objects in the two disciplines manifest, it is well likely that the number of discipline-specific phenomena largely exceeds the number of commonalities. As a result, all analogies which are proposed between the two disciplines should be rigorously checked, and methods should never be blindly transferred but always carefully adapted to the specific needs of the target discipline [55]. Object-based analogies bear a high risk of overstating similarities in interdisciplinary research and may easily lead to wrong conclusions and inadequate transfer of methods and models. Schleicher, for example, compared languages with organisms and derived from this comparison the hypothesis that languages would also grow old and die [33, 59]. To circumvent this problem we propose to concentrate on analogies between *processes*. *Process-based analogies* (PBA) are explicitly agnostic regarding further analogies between the research objects themselves. In taking processes as our starting point, we build on general approaches to analogy, which usually claim that the core of analogy are similarities of functions [69]. Focusing specifically on processes rather than functions is justified by the evolutionary background of biology and linguistics: processes serve as the major *explanans* in evolutionary research. Identifying analogies between evolutionary processes in these two fields as different as biology and linguistics may thus contribute to a unifying explanatory framework of evolutionary processes. Even when basing analogies on processes, however, we should not forget that we are dealing with very different disciplines, and any methodological transfer should be accompanied by a careful adaptation of methods to the needs of the target discipline. Future research will need to decide whether we the proposed analogies reflect general evolutionary processes or processes specific to the respective disciplines. Our uncertainty regarding the extent to which a unification of evolutionary processes in biology and linguistics is possible is reflected in Fig. 2, where we have marked the degree by which the processes in the disciplines overlap with a question mark.

The focus on processes produces potentially fruitful novel analogies. It can also identify processes that seem to be exclusive to one of these two historical sciences (Fig. 2). Among the exclusively linguistic processes, we identify such processes as *sound change* (Fig. 2:14), *semantic change* (Fig. 2: 16), or *purification* (Fig. 2: 10). Neither of these processes seems to have a biological counterpart: It has been proposed to compare sound change in linguistics with concerted evolution in biology [67], but we think that the analogy between the two processes does not completely hold. In concerted evolution, two traits change in a similar manner. During sound change, the phoneme system of a language changes [70]. An analogous process in biology would be a process in which the canonical amino acids constantly changed during evolution. During semantic change, the associations between words and concepts are restructured ([55], pp. 24–27). One might think of comparing this with changes in the regulation of genes in a genome which may yield drastic changes in function [71]. However, while biological function is still determined and restricted by the nucleic and proteic forms, no necessary limits are imposed on the association between forms and meanings in natural languages: the association is arbitrary in the sense that a substantial link between form and meaning in languages is not necessary [72, 73]. Purification is a process by which language change is actively triggered with the goal to preserve the pure state of one’s mother tongue. One paradigmatic example for this kind of change is the Romanian language which was heavily influenced by neighboring Slavic varieties, until, around the end of the 18th century, nationalist movements triggered a purification process by which Slavic loanwords were successively replaced with native Romance words [74].

Exclusively biological processes include, among others, asexual (Fig. 2:6) and sexual reproduction (Fig. 2:12), but most likely also natural selection in a strict sense (Fig. 2:9). Some scholars claim that there is evidence that certain aspects of languages, like their sound systems, correlate with environmental factors [75], while other aspects, like their morphological complexity or the way they change, correlate with demographic factors [76, 77]. But languages are not independent of the ones who use them. They replicate via acquisition (of one’s first language, Fig. 2:4) and learning (of a further language, Fig. 2:1). Although we cannot exclude, that selection processes in biology and linguistics are similar and that a common theory of fitness could be derived [78], and that languages, for example, differ regarding the difficulty with which they can be learned, we think it would be premature to draw any process-based analogies here. Linguists tend to avoid the discussion of the fitness of languages due to its political and cultural implications, emphasizing that all natural languages are learnable within the normal time span that children need to acquire a language. There are also no known cases of languages becoming abandoned by their speakers due to their difficulty, since speakers always slightly adjust their languages to fulfil their communicative needs and thus maintain the functionality of their most important communication tool. Even if ease of transmission was a factor potentially influencing language evolution, as suggested by W. Ford Doolittle (the first reviewer of this manuscript), learning difficulty is by no means the sole factor that leads to language spread. The spread of English as a major second and first language, for example, was largely due to political factors, depending on those who carry the language rather than the language itself. It was not the rather simple grammatical structure of English that favored its spread but the fact that large powerful countries in different parts of the world use English as their first and official language. That the speaker size and especially the amount of second language speakers may have an impact on the way languages evolve is most likely [76, 77]. In order to be able to assess the various factors more substantially, however, much more research is required in the future, and we are careful in drawing any analogies with biological processes, as we still do not know enough about all the mechanisms involved in language evolution. For this reason, we are careful in identifying a direct counterpart process of natural selection in the linguistic world. There is ample evidence that some kind of selection occurs during language evolution [79, 80]. This selection is often called cultural selection, and we place it among the exclusively linguistic processes (Fig. 2:7).

The large amount of disciplinary-internal processes for which we could not find any counterpart is a challenge for current research in the evolutionary sciences, and a specific challenge for biologists and linguists. One the one hand, future research may show that some of these processes actually have counterparts in the other discipline, on the other hand, we may make progress in explaining *why* those processes are unique to a specific domain. In both cases, we will gain deeper insights into both the unity and the disunity of evolutionary processes across disciplines. But at least as important as the differences are the newly identified commonalities, which we will discuss in detail in the following section.

## New analogies for biology and linguistics

The PBAs which we identified can be roughly divided into three categories, depending on the type of process which is involved. Tree-like processes represent the classical Darwinian framework of descent with independent modification between lineages, like divergence, and drift. Introgressive processes represent a network model of evolution in which lineages can influence each other after divergence, be it lateral transfer and borrowing (Fig. 2:13), hybridization and creolization (Fig. 2:8), or protein assembly and word formation (Fig. 2:15). Systemic processes represent a systemic model of evolution in which the interdependence between the components of evolving objects has a direct impact on the way they change (Fig. 2:17).

### Biological methods can help to automatize the identification of homologous words

While the process of vertical descent is well established in both linguistics and evolutionary biology, it is notoriously difficult to define which words or other linguistic features are historically related across languages. Identifying words of common origin, for example, is of fundamental importance to compare diverging languages. In linguistics, the term *cognate* is used to address those words which share a common origin in which no lateral transfer occurred. So *cognacy* is, strictly speaking, not the same as *homology* in evolutionary biology [81], although it is often used interchangeably. Just like gene trees can be used to infer species trees in biology, sets of cognate words can be used to infer the relationships between languages [61, 82]. Problematically, the identification of cognates suffers from numerous practical limits. Traditionally, cognates are identified manually in linguistics, without any help of computational methods. But since the classical approaches to cognate identification are notoriously difficult to apply, the number of words used in phylogenetic language comparison is restricted to very small parts of the lexicon which are assumed to be neutral with respect to culture and present in all languages across all times. These basic parts of the lexicon, which are supposed to change slowly, only consist of about 200 words per language [83].

The overall number of words across languages varies drastically, and it is difficult to come up with a reliable statistics. However, given that near-native abilities of second language learners for the major European languages require the knowledge of about 4,000 to 5,000 words [84], it is obvious that cognate sets in computational applications cover an extremely restricted set of words. Despite this extreme restriction, only a fragment of the 7,000 languages spoken today have been thoroughly investigated. Given a large and increasing amount of digitally available data, the discipline can no longer be handled by manual inspection alone.

**Fig. 3:**
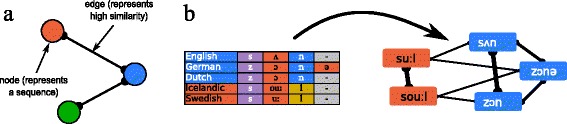
Sequence and word similarity networks. **a** In sequence similarity networks, sequences and similarities between sequences are represented in a network. Sequences are represented as nodes, and similarities between sequences are represented as edges if they exceed a certain threshold. Since evolutionary processes leave certain traces in the topology of these networks, they can be identified by applying standard network techniques. **b** Since words can be modeled as sequences of sounds, it is straightforward to create networks which represent the similarity among words. Due to the peculiarities of language evolution, however, similarity measures need to be specifically adapted to linguistic needs. As in biology, linguists start from alignments, as illustrated for words meaning ‘sun’ in five Germanic languages, but specific scoring functions are used

In evolutionary biology, the problem of identifying processes of vertical transmission in large amounts of data has given rise to a large collection of methods to deal with homolog identification. Some of these methods have already been successfully adapted to linguistic needs [50], thereby showing to biologists that their methods have an even larger application range than assumed by those who originally designed them. In order to enhance these methods further, *sequence similarity networks* could turn out to be very fruitful for historical linguistics (see Fig. 3). In biology, they can be used to identify highly divergent gene families [85]. When adapting the biological similarity scores used in sequence similarity network approaches to linguistic needs, similarity graphs could be used to search for highly diverse cognate sets across languages, and, potentially, even language families, expanding recent automatic approaches to search for deeper relationships among the more than 400 identified language families of the world [86].

### Incomplete lineage sorting as an introgression-free explanans for mosaic cognate patterns

**Fig. 4:**
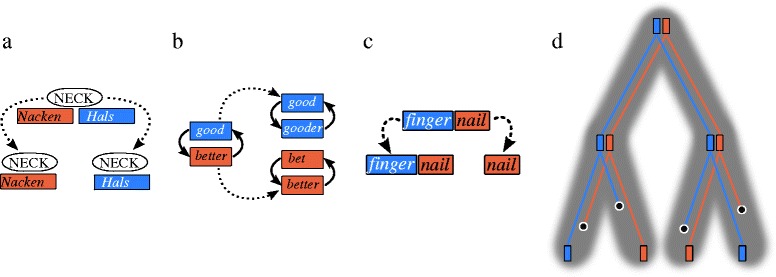
Polymorphisms in language evolution. **a** Synonymy: languages have many nearly synonymous words (German *Hals* and *Nacken* both mean ‘neck’ in English). They can be interchangeably used to express one and the same concept. Near synonymy is often resolved by dropping one of the two words. **b** Analogy: languages with complex morphology (case systems, etc.) often have irregular paradigms which consist of different stems (like *good*, *better*, *best* in English). These paradigms are often resolved retaining only one form and adapting the other forms to this model (e.g., *good*, *gooder*, *goodest*). **b** Derivation: words can be slightly modified by adding affixes (*word derivation*) or merging to words with each other (*compounding*). Often, both the modified or merged forms can still be interchangeably used with the original forms. They can also replace the original forms. **d** Incomplete lineage sorting: if rapid divergence occurs before the polymorphisms are resolved, they may yield patterns that seem to be in contradiction with tree-like divergence

Polymorphisms can create mosaic patterns of homologous genes, but also of cognate words. In linguistics, they may occur on various levels, depending on the data which is used to model language evolution (see Fig. 4). Mosaic patterns can be tentatively explained by introgression (concrete borrowings or language contact in general). In biology, however, another, introgression-free explanans is also commonly considered. This alternative explanans is *incomplete lineage sorting* (ILS, Fig. 2:5). In this process, ancestral polymorphisms are not fully resolved into lineages when rapid divergence occurs ([87], p. 351). ILS was, for example, used to account for the fact that 30 % of the human genes appear more similar to their homologs in Gorilla than to their homologs in Chimpanzee [88]. In the scholarly tradition of historical linguistics, there is no term that might serve as a counterpart. The process, however, is well-known, and was inherently already addressed when linguists like Johannes Schmidt (1843 – 1901) and Hugo Schuchardt (1842 – 1927) refuted Schleicher’s family tree theory of language divergence right after it was proposed [89–91]. As shown in Fig. 4, there are various sources for polymorphisms in language evolution. If polymorphisms created from word formation (see below) or lexical replacement are resolved after rapid divergence of the languages, ILS creates patterns quite similar to those observed with genetic alleles in biology. Importantly, phylogenetic methods in biology [92, 93] allow one to reconstruct a lineage tree (i.e. a species tree) taking ILS into account. Considering the ILS process and the associated methods could thus directly benefit linguistics. The Indo-European language family is a prominent example. Although the eight main branches of Indo-European are well established, and even the system of the proto-language is rather well understood, scholars have huge problems in determining the exact branching order of the eight groups. In the light of ILS, this may be less surprising. Recent studies on ancient genome-wide data of ancestral Europeans point to a rapid expansion of Indo-European languages in prehistorical times [94]. A careful investigation of the effects of ILS on language data may bring supporting evidence from linguistics.

### Network approaches shed light on introgressive processes in language evolution

**Fig. 5:**
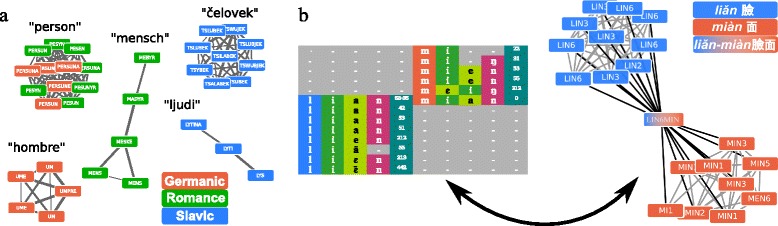
Similarity networks applied to linguistic data. **a** Similarity networks are reconstructed from global alignments for words meaning ‘person’ in Germanic, Romance, and Slavic languages (data taken from [101]). Five large connected components are identified. While three of them are homogeneous regarding the language family and show true cognate sets common in the respective branch of Indo-European, the top-left cluster contains words from all three branches. This cluster mainly shows Romance reflexes of Latin persona ‘person’. Slavic and Germanic words occurring in this cluster are all borrowed. **b** Similarity networks are reconstructed from local alignments for dialect words meaning ‘face’ in 20 Chinese dialect varieties (data taken from [132]). The data contains three variants, two simple words *liǎn* and *mián*, two words of different origin, and one fused form *liǎn*-*mián*. Numbers in the alignment reflect tone patterns, which are characteristic for South-East Asian languages. Edges colored in black differ in their local and global alignments, edges colored in gray show identical alignments for local and global analyses. The fused form serves as a hub connecting the two components. Data and code to reproduce the networks is available from the data and material accompanying this article (Additional file 1)

In addition to improving the explanation of the complexity produced when intellectual objects of linguistics undergo tree-like evolutionary processes (such as vertical descent or ILS), PBA could also help linguists in their struggles for handling introgressive processes. Introgressive processes are a constitutive part of language evolution. Borrowing of words, the PBA of lateral gene transfer [49–51] (Fig. 2:13), is very frequent and may effect more than 40 % of the stable parts of a language’s lexicon [95]. For the task of automatic borrowing identification in linguistic data, sequence similarity networks could again be useful. In biology they are increasingly used to study lateral gene transfer [96–98] and they could be employed in a similar fashion in historical linguistics, as illustrated in Fig. 5a.

Introgressive processes in language evolution are not restricted to processes like borrowing, in which two or more languages interact, but they can also occur in one and the same language. Words are often created from smaller meaningful units from the same language (morphemes) via processes of word formation [11]. Word formation can be roughly divided into two processes: derivation and compounding [99]. While compounding creates new words by merging existing ones, derivation uses affixes which cannot be used in isolation but only when being attached to other words (compare, e.g., the *-ness* in English *sick-ness*). Word derivation and word compounding result in the emergence of word families, that is, groups of words which are cognate within one and the same language. Word families play an important role in lexical organization: by decomposing words into smaller meaningful units (morphemes), speakers can quickly induce the meaning of words, even if they hear them the first time. As a result, speakers can understand between one and three times as many words as they know [100]. The size of word families can vary drastically, be it within one and the same or across several languages. The 60,000 words of the standard lexicon of German, for example, can be assigned to 8,000 word families comprising between 1 and 500 words [102].

The immediate consequence of word families is that cognate words across different languages are not necessarily completely cognate but may often exhibit different degrees of partial cognacy [81]. In Mandarin Chinese, for example, the regular word for ‘moon’, *yuè liàng*, consists of two morphemes, the first one originally meaning ‘moon’ in isolation, and the second one meaning ‘shine’ in isolation. In combination, they now mean simply ‘moon’. In Cantonese, the Chinese variety spoken in Hongkong, the regular word for ‘moon’ is *jyut*^6^*gwong*^1^, with the first morpheme being cognate with Mandarin *yuè*, but the second element, which means ‘light’ in isolation, being not cognate with the second element in Mandarin. Although methods for automatic cognate detection have been substantially improved over the last years [55, 103], none of the methods proposed so far is able to handle partial cognates across different languages. Word formation, especially word compounding, however, is very productive in many languages, especially in South-East Asian language families like Sino-Tibetan, Austro-Asiatic, Hmong-Mien, and Tai-Kadai ([104], pp. 62–67) which constitute more than 10 % of the worlds languages [105]. Compounding is not restricted to specific realms of the lexicon but also affects the core vocabulary of languages which is used in phylogenetic approaches. In the Chinese dialects, for example, about 50 % of all nouns and more than 30 % of all words in basic vocabulary are derived from fusion or derivation [106]. In biology, sequence similarity networks have been used to detect composite genes [107]. In a similar manner, word similarity networks could be used to automatically identify compound words, as illustrated in Fig. 5b. In a recent pilot study, it is further shown how a careful adaptation of similarity networks to linguistic needs allows to identify partial homologies (as the one between the Mandarin and Cantonese words for ‘moon’ shown above) with a high accuracy [106].

### Towards a new linguistics of proteins

**Fig. 6:**
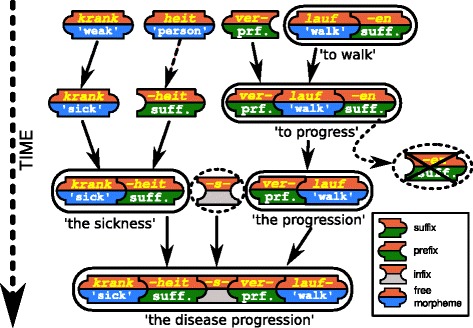
Word formation processes in the German language. Word derivation and word compounding are the major processes underlying word formation. Word derivation involves the combination of bound morphemes (suffixes, prefixes, and infixes) with free morphemes (regular words of a language). The graphic shows how the German *Krankheitsverlauf* ‘disease progression’ has been created in multiple stages by which the adjective krank ‘sick’ was nominalized with help of the suffix *-heit* and later compounded with the nominal form of the complex verb *verlaufen* ‘to progress’. Note that free morphemes may easily turn into suffixes during language change

In 2006, Mario Gimona proposed an analogy between the structure of proteins and the syntax of languages, necessitated by the higher complexity of “protein grammar” compared to “DNA grammar” [57]. This idea has been sporadically followed up in the biological literature, where the generation of new functions via the combination of different protein domains in biology is compared with the new meaning that languages produce by combining different words to new sentences [108]. The syntax of a language is usually understood as the set of rules needed to combine words to phrases and sentences which native speakers accept as well-formed examples which are “grammatically correct”. However, in linguistics, rule systems by which a set of elements are composed to create elements of a higher order are not restricted to syntax alone, but occur at various levels of organization [109]. There are phonotactic rules that handle the composition of sounds to form well-formed morphemes, there are morphological rules by which morphemes can be combined to form words, and there are even specific rules by which sentences can be combined to form texts [110]. If we take grammar as the cover term for any system of rules which transforms a set of symbols into a sequence of a higher order and function, the question for a grammar of proteins is where to draw the analogy with human languages exactly? Here, we think that a PBA between the process of word formation and the assembly of proteins [111], will be much more fruitful for evolutionary biology than the analogy between syntax and protein structure (see Fig. 6). While the syntax of human languages is extremely productive, being capable of creating virtually unlimited numbers of different sentences, the rules underlying word formation are much more restricted. Similar to protein evolution, only a small number of the theoretically possible words is ever realized in a language. Similar to proteins, the words which are realized can also be thought to form a single network of interrelated sequences [112]. A recent study on word formation in English and German further shows that the distribution of morphemes across words resembles the distribution of domains across proteins [113]. Although many aspects still require further research, major processes of word formation are well understood and have been investigated from multiple perspectives, including evolutionary [114] and cognitive aspects [115]. Especially automatic approaches to the unsupervised detection of morphemes date back to the 1950s [116], and many different methods have been proposed over the last decade [117–119]. A closer interdisciplinary exchange between biologists and linguists during which similarities and differences between the processes are identified might inspire new methods and models in *both* biology and linguistics. In biology, first attempts have been made to employ standard methods for natural language processing to study protein domain promiscuity [120, 121]. As these attempts were based on methods originally designed to analyze syntax in natural languages, shifting the methodological transfer to methods designed to analyze word formation might provide biologist with fresh and unexpected insights.

### Invoking a system-perspective to demystify the mysteries of language drift

Almost 100 years ago, Edward Sapir (1884–1939) made the strange observation that language change may produce strikingly similar phases after the divergence of lineages, independent of areal contact or environmental influence [122, 123]. Sapir called this phenomenon of convergence, seemingly conditioned only by common ancestry, drift. Up to today, a more thorough investigation of the phenomenon is lacking, and many linguists even discard it as a mystical observation [124]. If we look at the evolution of systems, that is, the evolution of interdependencies between components of evolving objects as yet another common process in biology and linguistics (Fig. 2:17), we find a possible explanans for this specific kind of language change. Evolutionary biologists distinguish two classes of interdependencies, depending whether they evolved neutrally (as in presuppression) or as a result of some selection. Typically, the evolution of several complex macromolecular machineries (such as the ribosomes or the splicesomes, [125] could be explained by a neutral increase of interdependencies between their elemental components, while convergences in regulatory networks (i.e. the fact that some patterns are more frequent than by chance, such as the feed forward loops in transcription networks) can be explained by considerations on the structure of these networks, e.g. the fact that sets of dependencies between elements stabilize or destabilize the function of the collective system that these elements form [71].

From a linguistic perspective, the use of the systemic perspective as an explanans for linguistic phenomena is by no means new. The structuralist movement, originally initiated by Ferdinand de Saussure (1857–1913) and later popularized by Roman Jakobson (1896–1982) was systemic in its core, assuming that ‘each system necessarily manifests as evolution, while, on the other hand, evolution necessarily bears systemic character’ ([126], p. 68). In historical linguistics, there is a large amount of literature on system-driven processes of language change. These include work on grammaticalization [127], direction in language change [128], and interaction between the varieties of one given language [129]. Likewise, it might be useful to consider ratchet-like (irreversible) processes which would affect linguistic systems in specific states, just as processes of constructive neutral evolution are assumed to affect biological systems [130]. The common change of languages which once diverged from a common ancestor is thus no longer mystical, but simply a consequence of the interdependencies which they inherited from their ancestor. It is more than likely that the many components of languages present interdependencies affecting their stability and rates of changes. For example, a recent use of sequence similarity networks on phoneme diversity across Chinese dialects revealed that phoneme diversity correlates with the grammatical classes to which these words belong [131]. Hence the internal grammatical structure of languages certainly affects their evolution. Unfortunately, the majority of investigations on interdependencies in linguistics is neither formalized nor quantified. investigations on interdependencies in linguistics is neither formalized nor quantified.

## Conclusion

We reported unities and disunities between evolutionary processes in historical linguistics and evolutionary biology. Common processes encourage the transfer of methods that had not been proposed earlier. The successful methodological transfer between the disciplines in the past encourages us to systematize the efforts of unification while at the same time being careful to not exaggerate the degree of similarity. Given the strong influence of biological approaches to quantitative research in historical linguistics in the past, the still low degree of quantification in historical linguistic research, and the new analogies which we proposed in this paper, it is clear that biologists may have an important role to play, given that their methods have a wider scope than anticipated earlier. On the other hand (following Schleicher’s idea proposed in 1863 [33]), given the amount and the subtlety of available historical documentation about the evolutionary processes that triggered linguistic diversity on earth, linguistic data could serve as an additional litmus test for the accuracy of biological methods, and biologists could profit from this advantage in detailed documentation.

In concrete terms, we showed, how biological methods can help to automatize the identification of homologous words in linguistics, how incomplete lineage sorting may serve as an introgression-free explanans for mosaic cognate patterns, and how similarity networks can be used to shed light on introgressive processes in language evolution. Furthermore, by refining the analogy of protein grammar, as a process-based analogy between the processes of protein assembly in biology and word formation in linguistics, both fields could profit from an interdisciplinary exchange and a deeper discussion of similarities and differences between the processes underlying the grammar of proteins and the processes underlying the grammar of words. The increasingly recognized need to account for the systemic dimension of evolution will likely prompt further unification across these fields and further interdisciplinary transfers. In the context of the theory of constructive neutral evolution, it may, furthermore, offer the long missing explanation for the mystical theory of parallel drift in the evolution of diverging languages.

Recalling that – apart from new analogies between evolutionary processes – we also identified processes which are specific to either biology or linguistics, it is important to keep in mind that the use of analogies should always be handled with great care. Not all evolutionary processes accounted for in one discipline necessarily need to have counterparts in other evolutionary disciplines, even if it is possible that future research will add process-based analogies where we failed to identify them. General evolution cannot be studied from within one discipline alone. Although unifying strategies can be fruitful, evolutionary explanations will remain fundamentally *pluralistic* since there is no reason to assume that all processes are common between biology and linguistics. In order to get a full picture of evolution, biologists and linguists need to complement their studies, trying to identify cross-disciplinary and discipline-specific evolutionary processes. If we want to understand how evolution triggered the diversity of substantial and intellectual objects on earth, we need to consider at least these two sister-disciplines.

## Reviewer’s comments

*We are very grateful to the reviewers for taking all the time to critically read our manuscript and to comment on it in their reviews.*

### Reviewer’s report 1: W. Ford Doolittle, Dalhousie University, Canada

I confess that I put off reviewing this because I feared that I would not understand it, or else would find it unoriginal: how could there be anything new to say about the similarities between historical linguistics and molecular phylogenetics? But I was wrong: I understand much of the paper and do think it says some important new things.

Basically what the authors propose is that we get even more serious about looking at the cross-applicability of methods and concepts being developed in linguistics and phylogenetics, particularly as these latter focus on evolutionary processes – rather than on the entities that evolve (words and proteins) – and also pay attention to the constraints that give direction to such processes such as syntax and molecular coevolution. Equally useful will be identification of processes that do not appear to be analogous between the domains. The authors suggest sound change, semantic change and purification as purely linguistic processes (the latter involving intent), and asexual/sexual reproduction and natural selection as purely biological.

It would be fun to argue about selection. The authors admit that there might be “cultural selection” (based on “egocentric”? or “content”? bias – see authors’ citation 70 [80]) that affect acceptance of certain elements within a language. Might it not also be that certain languages as systems are more likely to persist than others, either because of their ease of transmission (surely some languages are easier to learn than others) or affect on their speakers (surely language structure affects cultural “evolvability” somehow and unwritten languages have obvious limitations)? It may also be that in conceptualizing linguistic natural selection we should accept that evolution by natural selection can result from differential persistence as well as differential reproduction. Frédéric Bouchard (with whom the senior author has worked) has extensively developed this concept for biological evolution.

Authors make a number of observations which seem (to me, in my linguistic ignorance) novel, and well worthy of pursuit. For instance, applying models of incomplete lineage sorting (of alleles) to data in rapidly diverging languages seems a good idea, as does analogizing “the process of word formation in linguistics and protein assembly in biology”. It would be good to hear more about this and about using networks to identify composite words, as the senior author has already done for proteins (see their reference 94). It is also amusing that the numbers here are so close. Authors claim that there are about 200 universally conserved “basic parts of the lexicon”, and that second language learners need only master 4,000 – 5,000 words. There are maybe 200 universally conserved genes among all genomes, and the average prokaryotic genome has about 5,000 genes!.

Authors show a curious reticence to go all the way in analogizing language and genome evolution. They consider languages to be special since they are ‘products of the human mind’ and note that “If there was no speaker of the English language, a book containing Shakepeare’s Hamlet would just be a collection of paper with ink blots”. Actually, probably not. Surely clever Mandarin- (or even Martian-) speaking cryptographers could make some sense of the blots. And anyway, it’s analogously true that the sequence of bases in the human genome would only be just a sequence of bases without all the evolved machinery of gene expression and environmentally-affected epigenetic baggage, as opponents of genetic reductionism correctly but so tediously insist.

Authors’ response: *We thank the reviewer a lot for the summary. We are glad that despite the initial reservations of the reviewer our manuscript turned out to be comprehensible enough, also for those who are not experts in the field of linguistics. The reviewer mentions that it would ‘be fun to argue about selection’ in the linguistic domain, pointing to the possibility that persistence of languages is linked to the ‘ease of transmission’ or ‘affect on [...] speakers’. Although in preparing the manuscript, we talked a lot about this issue in our interdisciplinary team, we decided to cut it short in the paper, given not only the difficulty to exhaustively grasp the forces at work in language evolution but also due to the heat with which the topic is discussed in linguistics. We refined the relevant passage by adding some further reasons why we are still careful in drawing the analogy, concluding, that in order to be able to assess the various factors triggering “cultural selection” more substantially, much more research is required in the future. Nevertheless, we agree with the reviewer that it would be very interesting to follow up these questions in more detail and we hope that our paper encourages researchers from different disciplines to increase their interdisciplinary work, looking for solutions to this and other problems related to language evolution. We have slightly modified the relevant passage in the main manuscript, trying to take the reviewer’s suggestions more closely into account.*

*Regarding the proposed process-based analogy between word and protein compounding, the reviewer further mentions that it ‘would be good to hear more about this and about using networks to identify composite words, as the senior author has already done for proteins’ [*107*]. As a matter of fact, we have, while waiting for the reviews of this manuscript, managed to carry out some more detailed pilot studies along these lines, and a manuscript with the title ‘Using sequence similarity networks to identify partial cognates in multilingual wordlists’ has been accepted for publication in the “Proceedings of the Association of Computational Linguistics 2016 (Short Papers)”. In this study, which would have gone beyond the scope of the current paper, we show how a careful adaptation of sequence similarity networks to linguistic needs allows us to identify partial homologies in linguistic datasets with a high accuracy [*106*]. We have now modified the manuscript in such a way that we directly mention this study along with a brief example, thus showing that similarity networks can indeed successfully be used to detect homologies across compound words in different languages.*

*As a final point, the reviewer mentions, with a certain regret, that we ‘show a curious reticence to go all the way in analogizing language and genome evolution’, which is definitely correct, but not necessarily since we ‘consider languages to be special’, but more since our experience with parallels proposed between the two fields in the past has led us to be rather cautious. In earlier work on the development of the family tree model in the discipline of linguistics, in which the first author was involved [*91*], it could be shown that – in contrast to the conviction of many scholars – it was an independent development in both disciplines, evoked by the emerging paradigm of uniformitarianism that triggered the development of the tree model rather than interdisciplinary transfer. One could thus argue that – if only the processes are strikingly similar – scholars may sooner or later come up with similar ways to handle them, with or without analogies drawn between disciplines. On the other hand, many of the analogies that were proposed so far, be it the one between languages and organisms by August Schleicher that was mentioned earlier in the manuscript, or the recent one between sounds in languages and nuclein bases in biology, turned out to be disappointing, unfruitful, and at times even completely wrong. While holding back ourselves, we hope, nevertheless, that our idea to start from common processes when searching for potentially fruitful analogies will offer us and our colleagues a tool to channel future methodological transfer across different disciplines. Furthermore, the reviewer has convinced us that our statement that Shakespeare’s work would ink blots on paper if there were no speakers of the English language to read it was essentially ill-chosen, not serving the point we wanted to underline, namely, the fact that the medium in which the research objects are realized differs largely in biology and linguistics, and that – in contrast to biology – the aspect of transmission via learning represents a different process of replication and manifestation. We therefore deleted the sentence from the manuscript.*

### Reviewer’s report 2: Eugene V. Koonin, NCBI, NLM, NIH, USA

#### Reviewer summary

The article by List and colleagues draws multiple analogies between evolutionary processes in biology and linguistics. To me, all, rather numerous articles and a few books that I have read on comparisons between biology and linguistics share the same, rather regrettable aspect: they seem very attractive and enticing to begin with but then, disappoint rather sorely. Regrettably, the present article is no exception. Quite frankly, I find that the title of the paper [original title: “Explaining evolution in biology and linguistics using common processes”, note by the authors] is a misnomer: nothing is explained here neither in biological evolution nor in the evolution of languages.

I agree that the ’process-based analogy’ touted by the authors makes more sense than the (apparently, more traditional) object-based analogy. I can also accept that there is substantial ILS in linguistic evolution and that there is some logic in the analogy between protein folding and word formation. The problem is that, as a student of biological evolution, I cannot formulate the new perspectives or ideas that I get from this article. Sadly, I think that I learned nothing truly new and substantial except for some details on the history of evolutionary linguistics and the interactions between linguists and biologists, in particular Schleicher and Haeckel (these historical details are fascinating). I cannot rule out that linguists do get something fresh out of this but the article has been submitted to a biology journal, so one could expect there to be something biologically relevant and perhaps interesting.

Authors’ response: *We thank the reviewer very much for his critical review. First, we agree that the title may have been ill-chosen and changed it accordingly in order to reflect more clearly the scope and content of the manuscript. The new title “Unity and disunity in evolutionary sciences: Process-based analogies open research avenues for biologists and linguists” hopefully gives a much clearer emphasis on what we wanted to discuss in the paper, namely that we face common and distinct processes in the evolutionary sciences, and that a focus on common processes rather than similarities in objects might help better in identifying fruitful analogies between disciplines which may eventually open new possibilities for future research.*

*Second, regarding the reviewer’s disappointment that while showing potentially interesting possibilities of methodological transfer from biology to linguistics, we do not offer ‘something biologically relevant and perhaps interesting’, we think it is important to emphasize that the scope of this paper regards evolution in general. What we want to show is that neither linguistic nor biological evolution are reducible to one another, even at the level of their processes. Therefore, understanding evolution requires (at least) these two complementary fields, which means that the lessons from biological evolution (and from historical linguistics) will never be self-sufficient to account for what an evolutionist ultimately cares for: evolutionary diversity. As biologists, we are compelled to work closer with linguists if we want to learn about aspects of evolution that are simply – and will otherwise remain – foreign to us. That is one lesson: our biological models are incomplete to account for evolution in general, so it would be not only unfortunate but also wrong-headed to forget about linguistic evolution in our accounts of the history of life. Biology Direct could almost have a section for issues related to evolution in general. As for the linguistic perspective, we have shown that in addition to the biological methods for phylogenetic reconstruction which are now regularly applied by historical linguists, there are many more potentially fruitful analogies which could give rise to methodological transfer (such as lessons from incomplete lineage sorting and sequence similarity networks). So linguists should and usually do care for evolutionary biology. But even if it might not yet seem obvious why linguistics might become methodologically relevant for biologists, we should not forget that quite a few methods have already been transferred from linguistics to biology, especially from the disciplines of computational linguistics and natural language processing [*43*]. Not only classical models of formal grammar (following the hierarchy of the linguist Noam Chomsky [*40*]) are used by biologist, but also advanced models like tree adjoining grammar, which can be used for RNA structure prediction [*44*], or inherently linguistic methods for document prediction which can be applied in protein classification [*45*], or stochastic analyses of syntax, being applied to study protein domain promiscuity [*121*]. In order to substantiate this claim, that – despite the many disappointing examples of failed analogies – there are examples for methodological transfer in both directions which could be labelled success stories, we have added further references and elaborated the details in the text.*

*To summarize, we hope that readers will get at least two major ideas from this work: (a) it makes sense to embrace a less biology-centered perspective on evolution in evolutionary studies (that is our ignorabimus); (b) introgressive processes are fundamental to make sense of both linguistic and biological change, so a network perspective constitutes, despite the dissimilarity between both fields, the broadest and most fruitful deep commonality to achieve a form of systemic unification. There is a common core of processes between biology and linguistics, which is why evolutionary biologists and linguists should care about each other’s findings. Overall, however, it is true that for all evolutionary sciences such systemic, process-based unifications will remain incomplete. Evolutionary sciences will remain pluralistic in methods and concepts, and another type of unification, i.e. operating in a piecemeal fashion and preserving the singularities of both evolutionary disciplines, will be needed to speak of evolution in general.*

#### Reviewer recommendations to the authors

The authors themselves notice that in the early days of genetics, and molecular genetics in particular, linguistic analogies and metaphors have been quite common. Some of these indeed became integral to the molecular biology lingo (transcription, translation), some are used much more sparingly (word, grammar), others have gone practically out of use (suffix, prefix, flexion). Regardless, though, why do these analogies do not really go beyond metaphors? Somehow it appears to me that this is not for the lack of effort on part of those interested in the linguistics-biology comparison. I feel that there is some deep disparity that precludes any substantial cross-fertilization. And here lies my major dissatisfaction with this paper. The problem is not that List et al. fail to find truly productive analogies between linguistics and biological evolutionary processes: many have tried and (at least, in my opinion) they all failed. The regrettable aspect of the paper is its rather careless but baseless optimism. I think the article would have been much improved if the authors embarked on a true critical discussion of these analogies and the reasons they do not appear to come across as genuinely fruitful.

Authors’ response: *We agree with the reviewer that many largely disappointing analogies have been drawn between both disciplines, and it is for this reason that we have showed what reviewer 1 called a ‘curious reticence to go all the way in analogizing language and genome evolution’. There is a deep dissimilarity between evolutionary biology and historical linguistics, even at the level of processes. There is nonetheless a possiblity of substantial cross-fertilization between both fields, especially around introgressive processes and network-like evolution, and as we can see from the application of formal grammars in biology (mentioned above) and the recent popularity of phylogenetic methods in linguistics, fruitful transfer of methods and models has already taken place in the past and in both directions. Currently, the direction of transfer goes especially from biology to linguistics, and this means that linguists import methods and concepts from biology, adapting them to their needs. Given the rapid growth of computational research in the area of natural language processing, however, it is by no means sure that the situation will always remain as this, and it might well be that even in the nearer future our proposed analogy between word compounding and protein assembly offers biologists who study linguistic approaches and patterns new insights into the phenomena in their discipline. Future will tell whether this claim is careless optimism, or whether exploiting common processes between linguistic and biological evolution will not only turn out to be fruitful but potentially also inspire cross-disciplinary research on a larger scale. But even if our optimism turns out to be unjustified, it will essentially contribute to our understanding of evolutionary processes if we can further narrow down the exact ratio of unity and disunity in the evolutionary sciences.*

*Nevertheless, we understand that we might have been exaggerating our optimism, and we have tried to trim it down to a level which is hopefully acceptable for the reviewer. First, we changed Fig.**2**to reflect more closely that the amount of common processes is presumably much smaller than the general amount of processes (we also try to indicate our own uncertainty by showing a scale with a question mark as value). We also modified the manuscript in several passages to reflect justified scepticism more closely, and we also added references that further substantiate the reviewer’s scepticism.*

#### Minor issues

In what sense did Watson and Crick ’detect’ DNA? They did not even discover it, they built the correct structural model of DNA that allowed them to explain replication.

Authors’ response: *We agree and rephrased the sentence accordingly.*

## Abbreviations

ILS, incomplete lineage sorting; PBA, process-based analogies

## Additional file

Additional file 1 The supplementary material contains the data and source code needed to reproduce the analyses to retrieve the networks shown in Fig. 5. It can be downloaded at https://zenodo.org/badge/latestdoi/5137/lingpy/process-based-analogies. (PDF 16 kb)
