# Supplementary material for: Unity and disunity in evolutionary sciences: process-based analogies open common research avenues for biology and linguistics
Source: Biol Direct. 2016 Aug 20;11:39. doi: 10.1186/s13062-016-0145-2 (PMC4992195; doi:10.1186/s13062-016-0145-2)
Supplement: Supplementary file 1 — The supplementary material contains the data and source code needed to reproduce the analyses to retrieve the networks shown in Fig. 5. It can be downloaded at https://zenodo.org/badge/latestdoi/5137/lingpy/process-based-analogies. (PDF 16 kb) [file 13062_2016_145_MOESM1_ESM.pdf]

Supplementary Material and Source Code Accompanying the Paper “Unity and disunity in evolutionary sciences”

All data can be downloaded from GitHub at:

- https://github.com/lingpy/process-based-analogies

Additionally, an official release with a DOI is available at Zenodo:

- https://zenodo.org/badge/latestdoi/5137/lingpy/process-based-analogies
